# Anticonvulsant and analgesic activities of crude extract and its fractions of the defensive secretion from the Mediterranean sponge, *Spongia officinalis*

**DOI:** 10.1186/1475-2867-12-15

**Published:** 2012-04-11

**Authors:** Afef Dellai, Hedi Ben Mansour, Audrey Clary-Laroche, Monia Deghrigue, Abderrahman Bouraoui

**Affiliations:** 1 Unité de Recherche des Substances Actives Marines (URSAM), Laboratoire de Pharmacologie, Faculté de Pharmacie, Avenue Avicenne, Monastir, 5000, Tunisie; 2 Laboratoire de biotechnologie et Valorisation de Bio Géo Ressources (LBVBGR) Institut Supérieur de Biotechnologie, ISBST BioTechPole Sidi Thabet Université Manouba, Ariana, 2020, Tunisie; 3 Laboratoire de pharmacologie des agents anticancéreux U916 (INSERM), Institut Bergonié, Bordeaux, France

**Keywords:** *Spongia officinalis*, Anticonvulsant activity, Analgesic activity

## Abstract

This study progresses in the direction of identifying component(s) from the Mediterranean sponge, *Spongia officinalis* with anticonvulsant and analgesic activities. We investigated the efficacy of crude extract and its semi-purified fractions (F1-F3) of the defensive secretion from *Spongia officinalis* for their *in vivo* anticonvulsant activity using the pentylenetetrazole (PTZ) seizure model and analgesic activity using the writhing test in mice. Among the series the crude extract exhibited interesting analgesic activity in a dose dependent manner. Similarly the fraction F2 showed a partial protection of mice from PTZ-induced seizure and interesting analgesic activity in a dose dependent manner. The purification and the determination of chemical structure(s) of compound(s) of this active fraction are under investigation.

## Introduction

The inability to cure contemporary disease such as cancer, AIDS, arthritis, Alzheimer and the growing incidence of drug-resistant infection diseases have stimulated the need for the development of new drugs from natural sources. Since the few last decades, marine environment have been recognized to be a rich sources of bioactive metabolites with varied biological and pharmacological activities [1,2]. Covering around 70% of the planet surface, the oceans possess a huge potential for the new discovery often on novel molecules. The most interesting phyla with respect to pharmacologically active marine compounds include bacteria, fungi, algae, soft corals and gorgonians, sea hares and nudibranchs, bryozoans, tunicates and especially sponges [3]. Marine sponges have been considered as a gold mine during the past few decades with respect to the diversity of their secondary metabolites and continue to provide novel natural products with a remarkable chemical diversity. It is not surprising that a sponge natural product possessed different pharmacological properties such as ceramide from *Negombata corticata*[4] which displayed anticonvulsant activitiy; manoalide, a sesterpenoid compound, from *Luffariella variabilis* which displayed anti-inflammatory, analgesic and antibacterial activities [5,6]. The objective of the present study was to evaluate for the first time the potency of crude extract and its semi-purified fractions (F1-F3) of the defensive secretion from *Spongia officinalis* for inhibiting convulsion induced by PTZ and for inhibiting writhes induced by acetic acid and phenylbenzoquinone with the aim of identifying novel molecules with interesting and potentially useful pharmacological activities.

## Materials and methods

### Sample collection and preparation of the extract

The marine sponge, *Spongia officinalis* was collected from the Mediterranean Sea, in various areas of the coastal region of Tunisia, at a depth between 2 and 3 meters. The collected samples were cleaned by rising with sea water and distilled water and transported in cool box to the laboratory where they are kept in a refrigerator in distilled water for 24 h. Identification of specimen was carried out in the National Institute of Marine Sciences and Technologies, Salamboo, Tunisia.

The samples were defrosted before use and then filtered using some cotton wool followed by passage through a Whatman filter paper # 1. The filtrate was lyophilized to give the crude extract of the defensive secretion. The powdered extract was stored at −20°C until use.

### Purification of the crude extract of the defensive secretion

Often, bioactive compounds constitute a very minor part of the crude extract. In order to localize the active fraction, extract of the defensive secretion of *Spongia officinalis* was purified, using C_18_ cartridges (Sep-pack, Supelco), by gradient elution with methanol–water mixture (0%, 25%, 50% and 80% methanol) to give 4 fractions (F0-F3). Methanol solvent was removed from fractions recuperated using rotating evaporator at 35°C and distilled water was then added to the residues and the aqueous phases were lyophilized. The powdered fractions were stored at −20°C until use.

Crude extract and fractions were diluted to the desired final concentration immediately prior manipulation.

### Animals

Swiss mice (20–30 g) of both sexes, provided from Pasteur institute (Tunis, Tunisia) were used. Animals were fed a standard diet ad libitum and allowed free access to drinking water. Housing conditions and *in vivo* experiments were approved according to the guidelines established by the European Union on Animal care (CEE Council 86/609).

### Anticonvulsant study in mice

Anticonvulsant activity was assessed according to the method described by Vogel [7]. Swiss mice of either sex (20–30 g) were used. Animals were divided into three groups of six mice each. Group one served as control and was treated with 10 ml/kg of saline by subcutaneous injection (s/c), the second group was given phenobarbital (120 mg/kg) (s/c) as a reference drug, and the third group was treated with the crude extract of the defensive secretion from *Spongia officinalis* (100, 200 and 400 mg/kg) and its semi purified fractions (F1-F3) at 200 mg/kg (s/c), 30 min before the intraperitoneal (i.p.) injection of pentylenetetrazole (PTZ) (90 mg/ml). The time taken before the onset of clonic convulsions, the duration of clonic convulsions, and the percentage of seizure and mortality protection were recorded. These parameters were compared in treated animals with those of control animals, in order to assess the anticonvulsant activity.

### Nociceptive tests

#### Acetic acid writhing test in mice

The analgesic activity was performed according to the method of Koster et al. [8]. Swiss mice (20–30 g) were selected one day prior to each test and were divided into three groups of six mice each. One group served as control (saline 10 ml/kg) (s/c). The second group was given the lysine acetylsalicylate (ASL) (200 mg/kg) by the same route, as a reference drug. The remaining group was treated with the crude extract of the defensive secretion from *Spongia officinalis* (100, 200 and 400 mg/kg) and its semi purified fractions F1, F3 at 200 mg/kg and F2 (50, 100 and 200 mg/kg) (s/c). All animals received 10 ml/kg (i.p.) of 1% acetic acid 30 min after treatment. The number of writhing was recorded during 30 min commencing 5 min after the acetic acid injection. A writhe is indicated by abdominal constriction and stretching of at least one hind limb.

#### Phenylbenzoquinone (PBQ) writhing test in mice

Swiss mice (20–30 g) were used. In this test the same procedure was used as described above, but writhing was induced by i.p. injection of 10 ml/kg of 0.04% PBQ in a 5% ethanol aqueous solution [9]. Antinociceptive activity was detected as a reduction in the number of abdominal constrictions exhibited by treated mice, with standard drug or with the sponge extract and its semi-purified fractions, as compared to the nociception control group, and was expressed as the percent of pain inhibition.

### Acute toxicity study

Eighty mice were divided into eight groups of ten animals each. One group served as a control and received 0.9% NaCl alone (10 ml/kg) given intraperitoneally, while the remaining seven groups were treated with increasing doses of the crude extract of the defensive secretion from *Spongia officinalis;* 300, 500, 700, 800 and 1000 mg/kg (i.p.), respectively. The mortality rate within a 24 h period was determined and the LD_50_ was estimated according to the method described by Miller and Trainter [10]. According to the results of acute toxicity test, the doses of 100, 200 and 400 mg/kg were chosen for experiments.

### Statistical analysis

Data are presented as the mean ± standard error (s.e.m). Statistical analysis was performed using Student’s *t*-test. The significance of difference was considered to include values of *P* < 0.05.

## Results and discussion

Epilepsy is one of the most common serious neurological conditions. Seizures have traditionally been recognized as a symptom of abnormal neuronal synchronization, and until recently have been thought to be a result of aberrant synaptic communication [11]. The insufficient efficacy of modern anticonvulsive drugs used in clinical practice, as well as wide global distribution of epilepsy, makes the design of novel compounds very important [12]. Convulsive seizures induced by administration of blocker of γ-aminobutyric acid (GABA) receptor Cl^-^ channels PTZ (90 mg/kg) (i.p.) were used as experimental epilepsy model. The forbrain is involved in the expression of clonic seizures, whereas the activation of brainstem structures participates in the expression of the tonic component [13]. Single dose, intraperitoneal administration of PTZ (90 mg/kg) caused clonic convulsions as well as lethality in mice. The pretreatment of mice with Phenobarbital (120 mg/kg) completely prevented all manifestations of convulsive attacks (Table 1). Pretreatment of the mice with the crude extract of the defensive secretion of *Spongia officinalis* caused a partial protection against PTZ-induced convulsions as shown in Table 1. The dose of 400 mg/kg had no significant effect on number of deaths. However, it prolonged the onset of clonic convulsions from 180 to 600 seconds and decreased the duration of seizure (Table 1). After s/c administration of the three semi-purified fractions (F1-F3) obtained by fractionation of the crude extract of the defensive secretion, protection against PTZ-induced convulsions was revealed with fraction F2 in a dose-dependant manner. At the dose of 100 mg/kg, F2 significantly increased the latency of seizure and decreased the duration of seizure compared to control. The next higher dose (200 mg/kg) had similar effects; moreover it caused 30% protection against PTZ-induced lethality in mice (Table 1). The prolonged onset time of PTZ-induced convulsions suggested an inhibitory action of the central nervous system [14]. The majority of currently available antiepileptic drugs (AEDs) fall into one of two pharmacological classes, those that modulate neuronal voltage-gated sodium channels and those that modulate inhibitory GABAergic neurotransmission. While, small number of AEDs may exert their effects via an interaction with voltage-operated calcium channels [15].

**Table 1:** Anticonvulsant effect of the subcutaneous administration of crude extract and its semi-purified fractions (F1-F3) of the defensive secretion of *Spongia officinalis* in the PTZ model in mice

Treatment	Dose (mg/kg)	Onset of first clonus (s)	Duration (s)	Seizure protection (%)	Mortality protection (%)
Control (saline 10 ml/kg)	-	180.16 ± 53.66	15 ± 1.16	0	0
Crude extract	100	149 ± 35.77^ns^	12 ± 87.24^ns^	0	0
	200	251 ± 17.88 ^ns^	10.7 ± 3.57^ns^	0	0
	400	600 ± 0^**^	5 ± 2.68^**^	30	0
F1	200	251 ± 9.83^*^	18 ± 3.57^ns^	0	o
F2	50	247 ± 17.88 ^ns^	8.7 ± 1.97^**^	0	0
	100	800 ± 17.88^**^	4 ± 0.71^**^	20	0
	200	1200 ± 89.44^**^	1 ± 0.89^**^	50	30
F3	200	249 ± 10.73 ^ns^	13 ± 1.07^ns^	0	0
Phenobarbital (reference drug)	120	-	-	100	100

Values are expressed as mean ± s.e.m. *P < 0.01, **P < 0.001, ns: not significant. n = 6 animals.

Most antiepileptic drugs are known to have strong analgesic effects [16]. Moreover the available analgesic drugs exert a wide range of side effects and are either too potent or too weak; the search for new analgesic compounds has been a priority of pharmacologists and pharmaceutical industries [17].

Among the several models of visceral pain, writhing test has been mostly used as a standard screening method [18]. In this study, results of the writhing tests are shown in Tables 2 and 3. The mouse writhing model involves different nociceptive mechanisms, such as the sympathetic system (Biogenic amines release), cyclooxygenases (COX) and their metabolites [19] and opioid mechanisms [20]. Acetic acid acts indirectly by inducing the release of endogenous mediator, which stimulates the nociceptive neurons sensitive to NSAIDs (non-steroidal anti-inflammatory drugs) and/or opioids [20]. The PBQ-induced writhing response is believed to be produced by the liberation of endogenous substance(s), notably metabolites of the arachidonic cascade [20]. However, the PBQ test is not specific for weak analgesics such as the NSAIDs, as it also detects centrally active analgesics [9,21]. The subcutaneous administration of the crude extract of *Spongia officinalis* (100, 200 and 400 mg/kg) produced a significant reduction in the number of abdominal constrictions throughout the entire period of observation in a dose related manner with respectively 49.19, 52.63 and 62.34% in the acetic acid writhing test (Table 2) and 63.24, 68.37 and 75.21% in the PBQ writhing test in mice (Table 3). Within the series studied, significant activity was observed with F2 (50, 100 and 200 mg/kg) in a dose related manner, with respectively 70.85, 77.53 and 85.42% in the acetic acid writhing test (Table 2) and 71.79, 79.48 and 82.05% in the PBQ writhing test in mice (Table 3), whereas at the same time, fractions F1 and F3 (200 mg/kg) inhibited writhing by 58.7 and 54.65% in the acetic acid writhing test and 69.23 and 70.94% in the PBQ writhing test in mice, respectively (Tables 2,3). Standard drug (ASL, 200 mg/kg) decreased the number of abdominal constrictions by 68.21% in the acetic acid writhing test and 74.35% in the PBQ writhing test in mice, respectively (Tables 2,3). We have demonstrated, using conventional pharmacological model, the analgesic property of the crude extract of the defensive secretion and its semi-purified fractions. The crude extract of the defensive secretion and its semi-purified fractions can induce antinociception by mechanism similar to non-narcotics and or narcotic drugs, perhaps by blocking the receptor or the release of endogenous substances that excite pain nerve endings [22]. NSAIDs such ASL produce their antinociceptive and anti-inflammatory action via inhibiting cyclooxygenases in peripheral tissues, thereby reducing PGE2 (prostaglandin E2) synthesis and interfering with the mechanism of transduction in primary afferent nociceptors [23]. This particular activity of the crude extract of the defensive secretion and its semi-purified fractions is probably related to their anti-inflammatory properties [24].

**Table 2:** Analgesic effect of the subcutaneous administration of crude extract and its semi-purified fractions (F1-F3) of the defensive secretion of *Spongia officinalis* in the acetic acid 1% writhing test in mice

Treatment	Dose (mg/kg)	Number of writhes ± s.e.m.	inhibition of writhing (%)
Control (saline 10 ml/kg)	-	82.33 ± 12.45	-
Crude extract	100	41.83 ± 12.52^**^	49.19
	200	39 ± 5.76^**^	52.63
	400	31 ± 3.46^**^	62.34
F1	200	34 ± 3.79^**^	58.7
F2	50	24 ± 2.09^**^	70.85
	100	18.5 ± 5.43^**^	77.53
	200	12 ± 4^**^	85.42
F3	200	37 ± 3.88^**^	54.65
Lysine Acetylsalicylate (reference drug)	200	26.16 ± 8.37^**^	68.21

Values are expressed as mean ± s.e.m. **P < 0.001. n = 6 animals.

**Table 3:** Analgesic effect of the subcutaneous administration of crude extract and its semi-purified fractions (F1-F3) of the defensive secretion of *Spongia officinalis* in the PBQ writhing test in mice

Treatment	Dose (mg/kg)	Number of writhes ± s.e.m.	inhibition of writhing (%)
Control (saline 10 ml/kg)	-	39 ± 2.6	-
Crude extract	100	14.33 ± 5.16^**^	63.24
	200	12.33 ± 4.8^**^	68.37
	400	9.66 ± 5.08^**^	75.21
F1	200	12 ± 3.74^**^	69.23
F2	50	11 ± 4.19^**^	71.79
	100	8 ± 3.4^**^	79.48
	200	7 ± 2.36^**^	82.05
F3	200	11.33 ± 4.32^**^	70.94
Acetylsalicylate of lysine (reference drug)	200	10 ± 2.6^**^	74.35

Values are expressed as mean ± s.e.m. **P < 0.001. n = 6 animals.

Evaluating anticonvulsant and analgesic properties in a single assay may not provide a full understanding of the actions of fraction or its utility. Further work to establish the active chemical constituent(s) of the active fraction F2 and ascertain its mechanism of action is currently going on in our laboratory.

Some known anticonvulsant and analgesic agents, such as ceramide [4], manoalide [25], disideine [26] and avarol [27] have already been isolated from other sponges. However, marine sponges often contain diverse and abundant microbial communities, including bacteria which are the most dominant group of microbial association in sponges, archaea, microalgae and fungi. In some cases, these microbial associates comprise as much as 40% of the sponge volume and can contribute significantly to host metabolism [28,29]. These data allows us to suggest that, in our case, the exact origin of the anticonvulsant and the analgesic properties remains unknown or from the active fraction of *Spongia officinalis* or from the symbiotic microorganisms. Isolation and cultivation of suspected symbiotic bacteria either from the surrounding sea-water or from the sponge could provide a better answer.

## Competing interests

The authors declare that they have no competing interests.

## Authors’ contributions

AD: made contribution to the study algogenic activities. HBM: Was responsible for the conception and design, testing and data acquisition, analysis and data interpretation and drafted the manuscript. Audrey C-L: made contribution to convulsing activity. MD made contribution to preparation of crude extract and its fractions of the defensive secretion from the Mediterranean sponge, *Spongiaofficinalis* Monia Deghrigue. AB made contribution to convulsing activity and statistical analysis. All authors read and approved the final manuscript.
